# Understanding relationships between asthma medication use and outcomes in a SABINA primary care database study

**DOI:** 10.1038/s41533-022-00310-x

**Published:** 2022-10-21

**Authors:** Marcia Vervloet, Liset van Dijk, Yvette M. Weesie, Janwillem W. H. Kocks, Alexandra L. Dima, Joke C. Korevaar

**Affiliations:** 1 Nivel, Netherlands Institute for Health Services Research, Utrecht, The Netherlands; 2 Department of PharmacoTherapy, -Epidemiology & -Economics (PTEE), Groningen Research Institute of Pharmacy, Faculty of Science and Engineering, University of Groningen, Groningen, The Netherlands; 3 General Practitioners Research Institute, Groningen, The Netherlands; 4 Observational and Pragmatic Research Institute, Singapore, Singapore; 5 Research on Healthcare Performance (RESHAPE), INSERM U1290, Université Claude Bernard Lyon 1, Lyon, France

**Keywords:** Asthma, Outcomes research

## Abstract

Adherence to inhaled corticosteroids (ICS) in asthma is suboptimal. Patients may rely more on their short-acting beta-agonist (SABA) to control symptoms, which may increase their risk of exacerbations and uncontrolled asthma. Our objective is to describe ICS adherence and SABA use among Dutch primary care patients with asthma, and how these are related to exacerbations and self-reported asthma control. Patients aged ≥12 years diagnosed with asthma who received ≥2 inhalation medication prescriptions in 2016 were selected from the Nivel Primary Care Database. ICS adherence (continuous measure of medication availability), SABA use (number of prescriptions), exacerbations (short courses of oral corticosteroids with daily dose ≥20 mg), and asthma control (self-reported with the Asthma Control Questionnaire; ACQ) were computed. Multilevel logistic regression analyses, to account for clustering of patients within practices, were used to model associations between ICS adherence, SABA use, and asthma outcomes. Prescription data of 13,756 patients were included. ICS adherence averaged 62% (SD: 32.7), 14% of patients received ≥3 SABA prescriptions, and 13% of patients experienced ≥1 exacerbation. Self-reported asthma control was available for 2183 patients of whom 51% reported controlled asthma (ACQ-5 score <0.75). A higher number of SABA prescriptions was associated with a higher risk of exacerbations and uncontrolled asthma, even with high ICS adherence (>90%). ICS adherence was not associated with exacerbations, whilst poor ICS adherence (≤50%) was associated with uncontrolled asthma. In conclusion, increased SABA use is an important and easily identifiable signal for general practitioners to discuss asthma self-management behavior with their patients.

## Introduction

Asthma is a common chronic respiratory disease affecting approximately 300 million people worldwide. It occurs in all age groups and is characterized by recurrent attacks of breathlessness and wheezing^1^. Treatment of asthma is usually a combination of controller and reliever medication. Controller medication, which is inhaled corticosteroids (ICS) with or without a long-acting beta-agonist (LABA), is needed to control the inflammation of the airways, whilst reliever medication, often short-acting beta-agonists (SABA), provides quick relief of asthma symptoms. A stepwise approach to asthma treatment is recommended by the Global Initiative for Asthma (GINA)^2^. Medication is stepped up (increasing the dosage of ICS, or ultimately adding biological medication or low-dose oral corticosteroids) if the current treatment is inadequate to control asthma symptoms. In recent years, these GINA recommendations have changed repeatedly as a response to emerging evidence on the effectiveness and use patterns of different treatments. For example, in 2016, GINA recommended starting with as-needed SABA, but due to insights on harmful effects of SABA-only treatment, as-needed low-dose ICS-formoterol is currently recommended as a first-step treatment^2^.

Although asthma can be effectively treated, many patients have suboptimal asthma control.

In a study among 8000 patients from 11 European countries, uncontrolled asthma was found in nearly half of the patients^3^. Asthma control comprises two domains: symptom control and reduction of risk factors. Uncontrolled asthma symptoms are an important risk factor for exacerbations. In addition, potentially modifiable risk factors are, amongst others, low ICS adherence, poor inhaler technique, high SABA use, and exposure to tobacco smoke or allergens. Poor asthma control increases the risk of exacerbations. Exacerbations may require hospitalization and have a negative impact on patients’ quality of life^4^. Moreover, a history of exacerbations increases the risk of future exacerbations^5^.

Adherence to ICS medication is often low^6–8^. The relationship between ICS adherence, use of SABA, and the risk of exacerbations is complex and studies have shown conflicting results. A systematic review showed that a higher level of ICS adherence was associated with a lower risk of asthma exacerbations, both in adults and children^9^. However, some observational studies concluded that higher ICS adherence levels were associated with increased use of reliever medication^10,11^ or even an increased risk of asthma exacerbations in children^6^. Differences in definitions and measurements of adherence and asthma control contribute to the complexity of this relationship.

Observational studies using administrative routine care databases offer the opportunity to study long-term variation in treatment use for large patient populations across long time intervals that resembles clinical practice^12^. A recent study using prescription data from the United Kingdom (UK) Optimum Patient Care Research Database prescription data showed that high SABA use predicted lower ICS adherence levels, but lower ICS adherence did not impact database-marked asthma control^13^. These real-life studies can give an indication of asthma management at the health system level at that time. Given the recent and frequent changes in asthma guidelines, examining treatment patterns using databases in different countries is needed to corroborate existing evidence and optimize treatment guidelines.

Up to now, no such study has been performed in the Netherlands. Therefore, the aim of the study is to describe ICS adherence and SABA use and their relationship with asthma outcomes (i.e., exacerbations and self-reported asthma control) in a large cohort of Dutch patients with asthma, using data from a large primary care database.

## Methods

The methodological recommendations for conducting and reporting observational studies using healthcare databases^14,15^ and the EMERGE guideline for conducting adherence research^16^ were followed. The TEOS framework was used to report the operationalization of adherence (more details in the Supplementary information)^17^.

### Study setting and population

Data were extracted from the Nivel Primary Care Database, which includes routine care data originating from electronic medical records from about 500 general practices (10% of all practices) across the Netherlands. These practices constitute a representative sample of the total population of Dutch general practices. Within the Dutch healthcare system, all residents are mandatorily registered with one general practitioner (GP), who coordinates their care. The database includes information on patient characteristics (age, sex), time-stamped GP consultations, diagnoses, and medication prescriptions. Diagnoses are recorded by GPs using the International Classification of Primary Care version 1 (ICPC-1). Prescriptions are coded using the Anatomical Therapeutic Chemical Classification system (ATC). Only coded data were extracted, not free text.

**Fig. 1:**
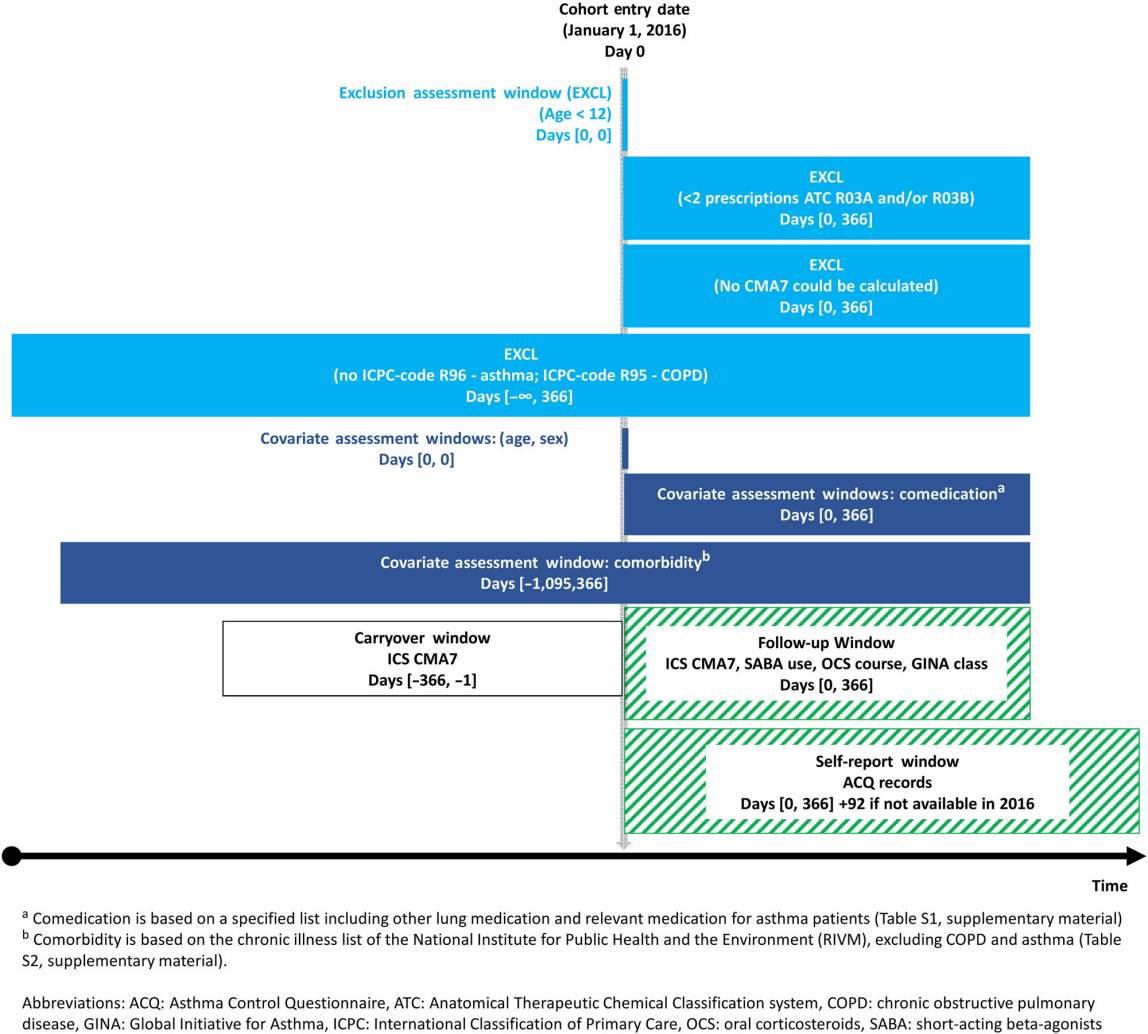
Study design and population.

**Fig. 2:**
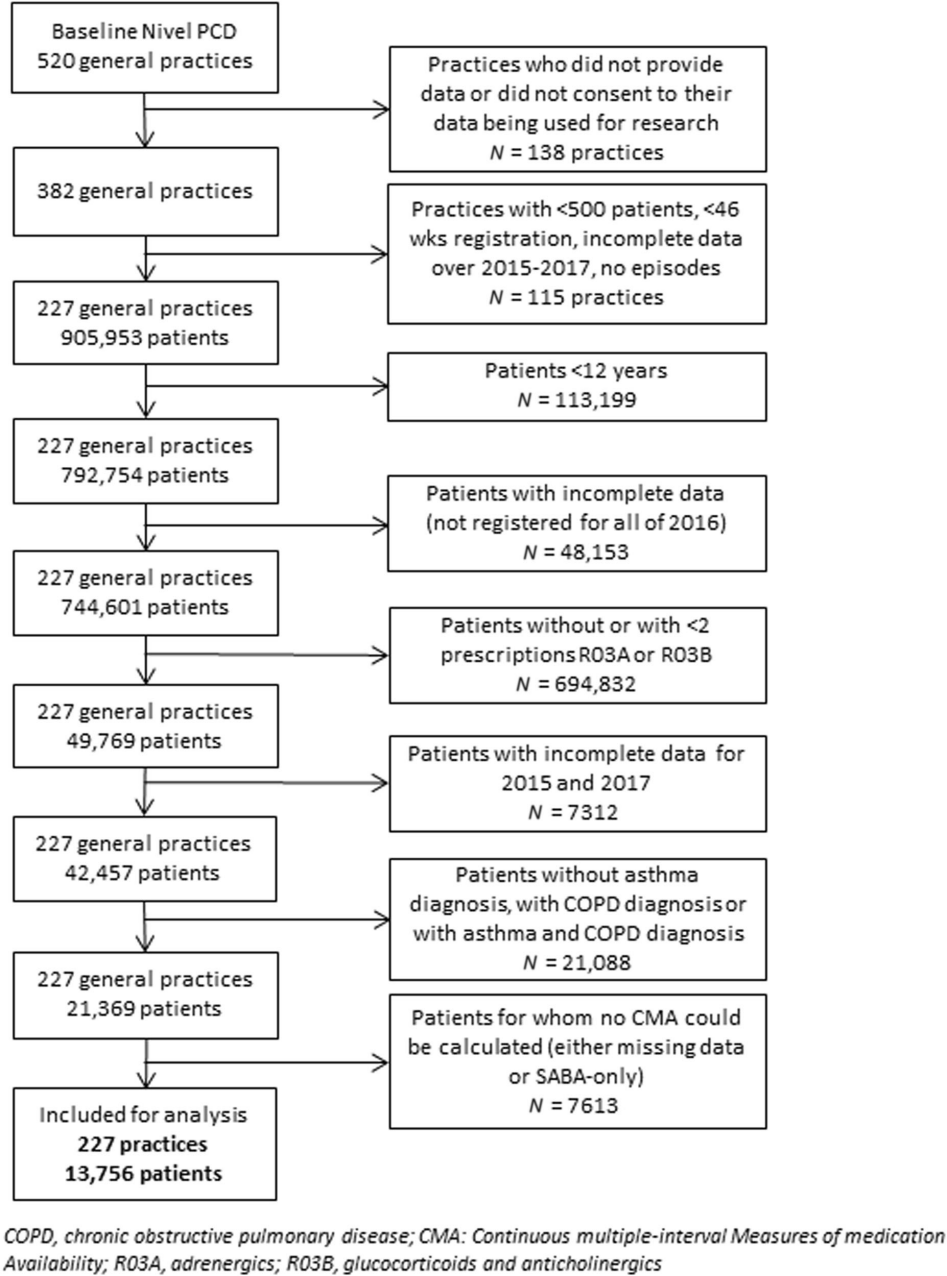
Flowchart of data preparation and selection steps.

These routine care data are registered by GPs for the primary purpose of providing continuous care to their patients, not for research purposes. Therefore, these data are not directly suited for research. It requires several data extraction and preparation steps to ensure high-quality data necessary for performing robust analyses for research purposes. For example, to minimize the occurrence of registration errors. These steps for this study included the following criteria per practice: a minimum of 500 patients (the average number of patients of 1 fte GP in the Netherlands is 2095), a minimum of 46 weeks of registration per year, complete data for the period 2015–2017 (necessary for adherence calculations), and illness episodes could be constructed (necessary for determining morbidities)^18^. As a result, a total of 227 general practices were included in this study, as they met these quality criteria. These 227 practices were spread throughout the Netherlands, in both urban and rural areas, and the age distribution of the patient population resembled that of the total Dutch population. Patients aged 12 years and older, diagnosed with asthma (ICPC-code R96) based on clinical history, physical examination, and supported by spirometry where available, who received at least two prescriptions of inhalation medication in ATC groups R03A (adrenergics) and/or R03B (glucocorticoids and anticholinergics) in 2016 were selected. Patients who were also diagnosed with chronic obstructive pulmonary disease (COPD, ICPC-code R95) in 2016 were excluded since the inflammatory process and the response to therapy in COPD is very different from that in asthma. Figure 1 graphically summarizes the study design and Fig. 2 is the flowchart of the study population.

### Ethics

This study has been approved according to the governance code of Nivel Primary Care Database, under number NZR-00318.050. Dutch law allows the use of electronic health records for research purposes under certain conditions. According to this legislation, neither obtaining informed consent from patients nor approval by a medical ethics committee is obligatory for this type of observational study containing no directly identifiable data (Dutch Civil Law, Article 7:458).

### Study outcomes

In adherence studies, it is important to distinguish between three temporal stages of adherence, being (1) initiation, whether the patient actually starts with the treatment; (2) implementation, whether the patient’s actual dosing corresponds with the prescribed dosing regimen; and (3) discontinuation, when the patient stops taking treatment before the end of the prescribed regimen^19^. These stages represent different types of behavior and subsequently require different approaches. This study focused on the quality of the implementation of patients’ prescribed regimens for asthma medication. A continuous multiple-interval measure of medication availability (CMA) was used to operationalize adherence, more specifically the CMA7. This operationalization, in contrast to CMA1 (known as the medication possession ratio) to CMA6 (with CMA3 to 6 known as variants of the proportion of days covered), takes into account carry-over from prescriptions, i.e., the CMA7 assumes that the medication is used as prescribed and oversupply from previous prescriptions is used first, followed by the new medication supply. CMA7 is calculated by dividing the number of days of theoretical use by the number of days between the start and end of the observation window (366 days in 2016). Days of theoretical use are calculated by extracting the total number of gap days (days for which no medication is available) from the total number of days of the observation window, accounting for carry-over for all prescriptions within and before this window. The CMA7 was expressed as a percentage (by multiplying by 100) and as a categorical measure using six categories: 50% or less; 51–60%; 61–70%; 71–80%; 81–90%; and 91–100%. Missing data on the number of prescribed doses for some prescriptions made it impossible to calculate prescription duration, which is necessary for adherence calculations. If the prescription duration could not be calculated for one of the prescriptions a patient received for ICS in 2016, this patient was excluded from the analyses. For this reason, 5873 patients were excluded, to improve the reliability and robustness of the data.

SABA use included prescriptions of salbutamol (R03AC02) or terbutaline (R03AC03). According to the GINA guidelines, patients with good asthma control should not need SABA more than twice a week (maximum of one to two SABA prescriptions per year)^2^. More than one SABA inhaler per month (thus 13 or more in a year) increases the risk of exacerbations and mortality^20^. Following these guidelines, SABA use was grouped into five categories: 0, 1–2, 3–6, 7–12, and 13 or more SABA prescriptions.

An exacerbation was operationalized as a prescription for a short OCS course, since the database did not contain information on hospitalizations or emergency room visits. An OCS course is defined as a prednisolone (H02AB06) or prednisone (H02AB07) prescription of at least 20 mg daily dosage for a respiratory disease (other than COPD) or if a tapering scheme was given. Patients who had no diagnosis linked to their OCS prescription were included in the analyses if there was no other known diagnosis for any of their OCS prescriptions. Two prescriptions issued within 14 days were considered as one OCS course.

Asthma control was determined based on self-report, with the Asthma Control Questionnaire (ACQ)^21^. This questionnaire contains seven questions, five to assess asthma symptoms over the past 7 days, one to assess daily use of reliever medication, and one question for the clinician to add the FEV_1_ resulting from the spirometry test. The sum score of the first five ACQ-items (ACQ-5) was calculated, leaving out the question on SABA use, since SABA use was included as a separate variable in our analysis. If more ACQ scores per patient were available over 2016, the last registered score in 2016 was included, since the aim was to relate ICS adherence over 2016 with the ACQ score. If no score was available in 2016, the ACQ score of the first quarter of 2017 was included, if available. Asthma control was dichotomized into controlled asthma (ACQ score <0.75) and uncontrolled asthma (ACQ score ≥0.75)^22,23^.

Relevant covariates included age, sex, comedication, comorbidity, and asthma severity. Age was grouped into 12–17, 18–39, 40–54, 55–64, and ≥65 years, based on patients’ age in 2016. Comedication was based on a specified list including other lung medication and relevant medication for asthma patients (Supplementary Table 1). The number of comedications (next to asthma medication) was grouped into 0, 1, 2, and ≥2 other chronic medications. Comorbidity was based on the chronic illness list of the National Institute for Public Health and the Environment (RIVM), excluding COPD and asthma (Supplementary Table 2). The number of comorbidities was grouped into 0, 1, 2, and ≥2 comorbidities. Asthma severity was operationalized as GINA class 1–5, according to the treatment steps recommended by GINA in 2016^24^, since our data were extracted over 2016. This categorization was based on the type and dose of medication (SABA-only for step 1, low-dose ICS for step 2, low-dose ICS/LABA for step 3, medium/high dose ICS/LABA for step 4, and add-on treatment for step 5) (Supplementary Table 3). Classification of patients was based on the prescription with the highest ICS dose a patient was prescribed in that year. For example, when a patient received three prescriptions in 2016, one of which was according to GINA treatment step 4, the patient was classified as GINA class 4.

### Data analyses

Descriptive analyses were performed to describe the sample. Differences between groups according to sex, age, comedication, comorbidity, and GINA class were tested with *χ*^2^ tests. A *p* value of <0.05 was considered to be statistically significant. Multilevel logistic regression analyses were performed to investigate associations between SABA use, ICS adherence, exacerbations, and asthma control, to take into account the clustering of patients within general practices. The analyses were adjusted for sex, age, comedication, comorbidity, and GINA class. For the association between SABA use, ICS adherence, and exacerbations, having one or more exacerbations compared to none was the outcome variable. For the association between SABA use, ICS adherence, and asthma control, having controlled asthma (ACQ score <0.75) compared to having uncontrolled asthma (ACQ score ≥0.75) was the outcome variable. Reference categories used in the analyses resembled the most positive scenario (i.e., 91–100% ICS adherence, 0 SABA prescriptions, 0 comedication, 0 comorbidities, and GINA class 2). Odds ratios with 95% confidence intervals were provided. An odds ratio >1 indicates a higher risk of having one or more exacerbations, and a higher chance of having self-reported controlled asthma.

### Reporting summary

Further information on research design is available in the Nature Research Reporting Summary linked to this article.

## Results

### Study population

Our sample consisted of 13,756 patients with asthma (Table 1). Six out of ten patients were female. About 7% of the patients were adolescents, whereas the largest age category was 40–54 years (28%), and nearly a quarter of the patients were 65 years or older. Most patients (88%) used other chronic medication besides their asthma medication and almost two-thirds of patients suffered from other chronic illnesses besides asthma. More than four out of ten patients were treated according to GINA step 4. ICS adherence averaged 62% (SD: 32.7). About 40% of patients had an ICS adherence level of 50% or lower, while almost a third had an adherence level of more than 90%. The majority of patients (86%) were issued two or fewer SABA prescriptions in 2016, and 14% had three or more prescriptions. The average number of SABA prescriptions was 1.2 (SD: 1.8). The majority of patients (87%) experienced no exacerbations.

**Table 1:** Characteristics of the study population of Dutch primary care asthma patients.

	Study population (*N* = 13,756)
	*n* (%)
Sex	
Male	5531 (40.2)
Female	8225 (59.8)
Age (years)	
12–17	981 (7.1)
18–39	2919 (21.2)
40–54	3800 (27.6)
55–64	2692 (19.6)
65+	3364 (24.5)
Comedication	
0	1618 (11.8)
1	2768 (20.1)
2	2978 (21.7)
>2	6392 (46.5)
Comorbidity	
0	4945 (36.0)
1	3146 (22.9)
2	2158 (15.7)
>2	3507 (25.5)
GINA class (*n* = 13,694)	
2^a^	1743 (12.7)
3	4886 (35.7)
4	6672 (48.7)
5	393 (2.9)
ICS adherence	
≤50%	5488 (39.9)
51–60%	1084 (7.9)
61–70%	1025 (7.5)
71–80%	990 (7.2)
81–90%	783 (5.7)
91–100%	4386 (31.9)
SABA prescriptions	
0	5916 (43.0)
1–2	5957 (43.4)
3–6	1600 (11.6)
7–12	233 (1.7)
≥13	50 (0.4)
Exacerbations	
0	11,947 (86.9)
1	1387 (10.1)
2	318 (2.3)
3	67 (0.5)
≥4	37 (0.3)

^a^GINA class 1 is not applicable, since patients in class 1 only use SABA. ICS adherence is not applicable to those patients.

Differences in patient characteristics between patients for whom an adherence rate could be calculated and those for whom it could not be calculated were small, indicating that this was rather due to registration errors in GPs’ information system than patient characteristics (Supplementary Table 4).

### Relation between ICS adherence and exacerbations

**Fig. 3:**
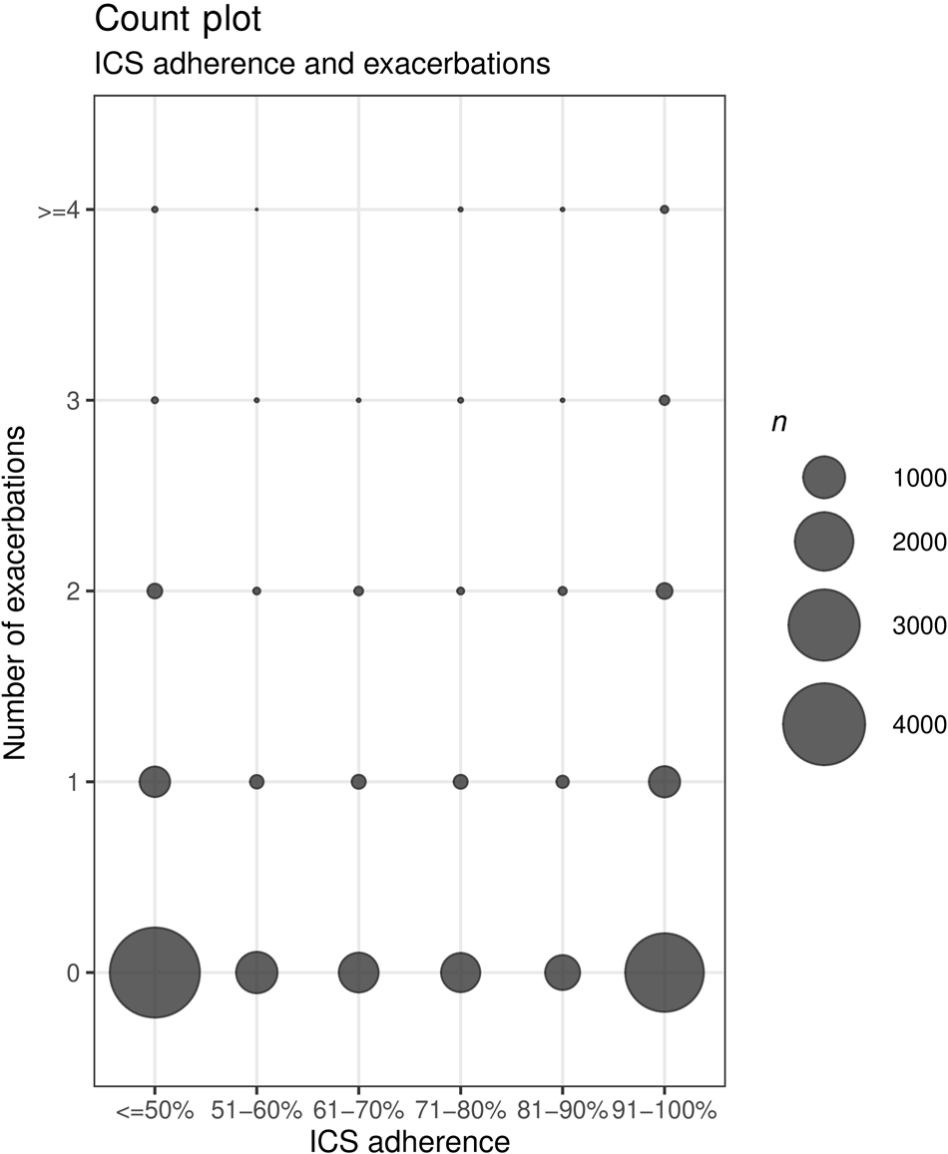
Countplot in which the number of exacerbations, defined as a short course of oral corticoid steroids (OCS), is shown per ICS adherence category for six categories.

No linear relationship was found between patients’ ICS adherence levels and the number of asthma exacerbations they experienced. Figure 3 illustrates that both patients with an ICS adherence level of 50% or lower and patients with an adherence level of more than 90% experienced exacerbations. Thus, exacerbations did not only occur in patients with poorer adherence to their maintenance medication.

### Relation between ICS adherence and SABA use

**Fig. 4:**
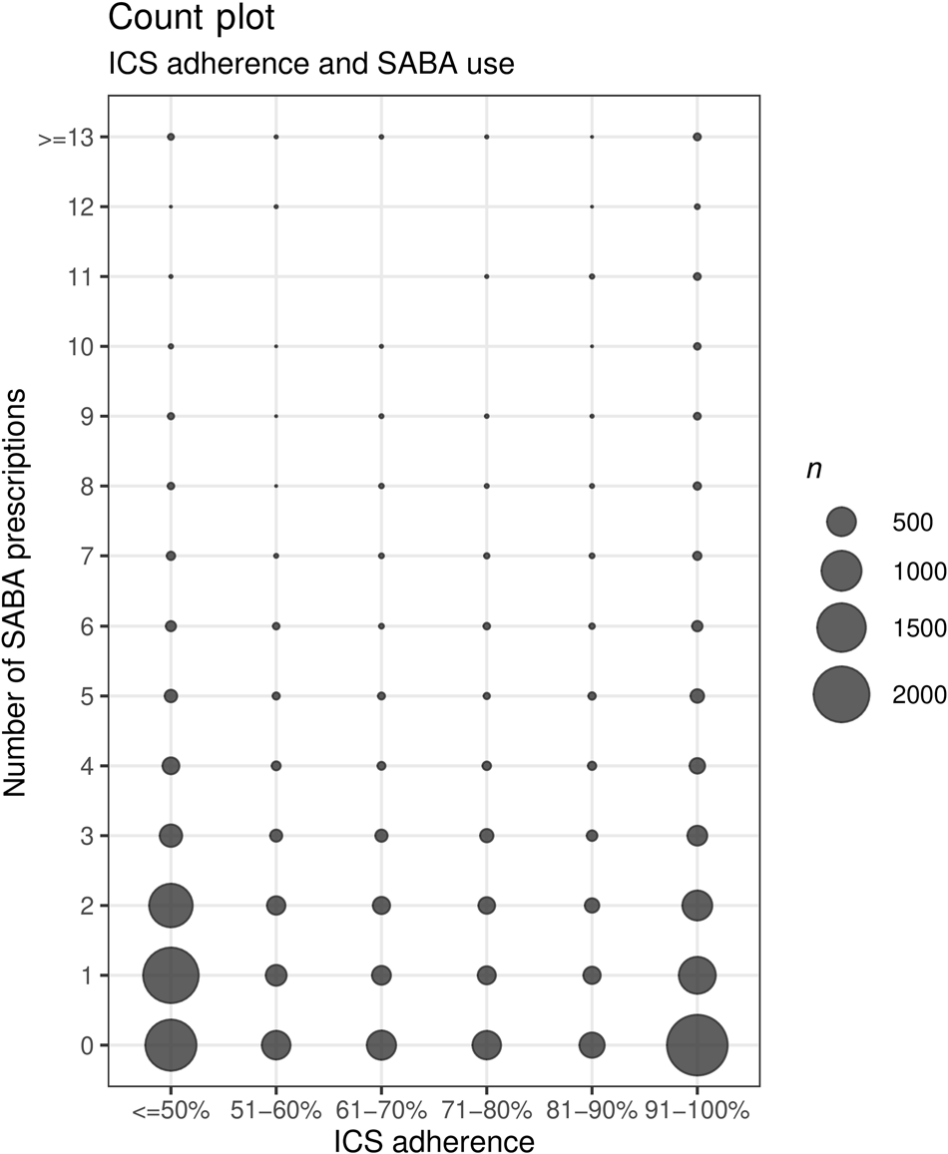
Countplot in which the number of SABA prescriptions is shown per ICS adherence category for six categories.

A U-shaped relationship was found between patients’ ICS adherence levels and the number of SABA prescriptions they were issued (Fig. 4). Although most SABA prescriptions were issued among patients with an ICS adherence of 50% or lower, patients with an adherence level over 90% also received more SABA prescriptions than patients with moderate (51–90%) adherence rates. Thus, SABA use is also higher among patients who are highly adherent to their controller medication.

### Factors associated with the occurrence of asthma exacerbations

In the multilevel analyses, it was investigated whether ICS adherence and SABA use were associated with having one or more exacerbations in 2016 (compared to none), whilst adjusting the results for patient and clinical characteristics. ICS adherence was not clearly associated with the occurrence of exacerbations. Patients with an adherence level of 51–60% and 71–80% had a significantly lower risk of experiencing exacerbations compared to patients with a high adherence level (over 90%). SABA use on the other hand was strongly associated with exacerbations; the odds of having one or more exacerbations increased with the number of SABA prescriptions (Table 2). Compared to using no SABA, even one or two SABA prescriptions were associated with a higher odds (OR 1.81, 95% CI 1.60–2.04) of experiencing an exacerbation. Patients receiving seven or more SABA prescriptions were three times more likely to experience exacerbations (OR 3.08, 95% CI 2.17–4.38) than patients with no SABA prescriptions. Being female, of older age, having (more) comedication, having comorbidities and more severe asthma (higher GINA class) all significantly increased the odds of having one or more exacerbations. There were minor differences between general practices in whether their patients experience exacerbations or not; about 6% of the variance (indicated by the intraclass correlation) was attributed to the general practice level.

**Table 2:** Results of multilevel logistic regression analysis showing whether ICS adherence and SABA use were associated with having one or more exacerbations compared to having none in the sample of 13,694 primary care asthma patients^a^.

	Odds ratio	95% Confidence interval	*p* value
ICS adherence (ref = 91–100%)			
81–90%	0.82	0.64–1.04	0.103
71–80%	0.79	0.63–0.99	0.038
61–70%	0.81	0.65–1.01	0.067
51–60%	0.69	0.55–0.88	0.002
≤50%	0.89	0.78–1.02	0.104
SABA use (ref = 0 prescriptions)			
1–2 SABA prescriptions	1.81	1.60–2.04	<0.001
3–6 SABA prescriptions	2.70	2.29–3.17	<0.001
7–12 SABA prescriptions	3.08	2.17–4.38	<0.001
≥13 SABA prescriptions	3.08	1.53–6.21	0.002
Sex (ref = male)			
Female	1.12	1.00–1.25	0.049
Age (years) (ref = 12–17)			
18–39	1.73	1.21–2.48	0.003
40–54	1.99	1.39–2.84	<0.001
55–64	2.00	1.39–2.89	<0.001
65+	2.36	1.62–3.43	<0.001
Comedication (ref = no comedication)			
1	2.42	1.73–3.39	<0.001
2	3.43	2.48–4.76	<0.001
>2	5.25	3.82–7.23	<0.001
Comorbidity (ref = no comorbidity)			
1	1.18	1.01–1.38	0.042
2	1.21	1.01–1.46	0.035
>2	1.20	1.00–1.44	0.049
GINA class^b^ (2 = ref)			
3	1.42	1.13–1.79	0.003
4	2.61	2.09–3.27	<0.001
5	5.00	3.64–6.85	<0.001
Random part	Coefficient	SE	
Between-practice variance	0.21	0.04	
	ICC (%)		
Practice level	6.1		

*ICC* intraclass correlation.

^a^Analyses controlled for sex, age, number of comedication, number of comorbidities, and GINA class, while taking into account that patients (*N* = 13,694) are nested within general practices (*N* = 195). An odds ratio >1 indicates a higher risk of exacerbations, compared to the reference category.

^b^GINA class 1 is not applicable, since patients in class 1 only use SABA. ICS adherence is not applicable to those patients.

### Factors associated with self-reported asthma control

For a subsample of 2183 patients, self-reported asthma control (ACQ-5 score) was available. Patients for whom an ACQ-5 score was available were older and more often had an ICS adherence level of over 90% than patients for whom this score was not available. Patients lacking this score more often had an adherence level of 50% or lower (Supplementary Table 4). About half of 2183 patients had controlled asthma, and 49.2% had uncontrolled asthma.

The results from the multilevel analysis, in which it was investigated whether ICS adherence and SABA use were associated with self-reported controlled asthma, adjusted for patient and clinical characteristics, showed that the more SABA prescriptions, the less likely it was that patients reported controlled asthma. Patients with one or two SABA prescriptions had low odds of reporting controlled asthma (OR 0.71, 95% CI 0.58–0.87), and patients being issued more than seven SABA prescriptions had an even lower odds (OR 0.14, 95% CI 0.05–0.38) than patients with no SABA prescriptions. Patients with a poor ICS adherence (50% or lower) were also less likely to have self-reported controlled asthma (OR 0.70, 95% CI 0.56–0.88). Women, patients with two or more other chronic medications and more than two comorbidities were less likely to report controlled asthma. Patients aged 65 years or older were more likely to report controlled asthma. Minor differences were found between general practices in whether patients reported controlled asthma, indicated by the amount of variance between practices being 3% (ICC) (Table 3).

**Table 3:** Results of multilevel logistic regression analysis in which we investigate whether ICS adherence and SABA use are associated with self-reported controlled asthma (ACQ score <0.75) compared to uncontrolled asthma (ACQ score ≥0.75) in the subsample of 2183 primary care asthma patients^a^.

	Odds ratio	95% Confidence interval	*p* value
ICS adherence (ref = 91–100%)			
81–90%	1.01	0.67–1.51	0.965
71–80%	1.04	0.71–1.53	0.822
61–70%	0.98	0.68–1.42	0.933
51–60%	0.72	0.51–1.01	0.059
≤50%	0.70	0.56–0.88	0.002
SABA use^b^ (ref = 0 prescriptions)			
1–2 SABA prescriptions	0.71	0.58–0.87	0.001
3–6 SABA prescriptions	0.42	0.31–0.58	<0.001
7–12 SABA prescriptions	0.14	0.05–0.38	<0.001
Sex (ref = male)			
Female	0.73	0.61–0.88	0.001
Age (years) (ref = 12–17)			
18–39	0.96	0.61–1.49	0.842
40–54	1.31	0.83–2.06	0.241
55–64	1.44	0.89–2.32	0.139
65+	1.67	1.02–2.73	0.042
Comedication (ref = no comedication)			
1	0.73	0.52–1.04	0.085
2	0.50	0.36–0.71	<0.001
>2	0.45	0.32–0.63	<0.001
Comorbidity (ref = no comorbidity)			
1	0.86	0.66–1.11	0.252
2	0.76	0.56–1.03	0.082
>2	0.55	0.40–0.76	<0.001
GINA class^c^ (2 = ref)			
3	0.97	0.71–1.32	0.837
4	0.67	0.49–0.91	0.012
5	0.63	0.30–1.31	0.214
Random part	Coefficient	SE	
Between-practice variance	0.10	0.05	
	ICC (%)		
Practice level	3.1		

*ICC* intraclass correlation.

^a^Analyses controlled for sex, age, comedication, comorbidities, and GINA class, while taking into account that patients (*N* = 2183) are nested within general practices (*N* = 128). An odds ratio >1 indicates a higher chance of having self-reported asthma control, compared to the reference category.

^b^No patients in this subsample have 13 or more SABA prescriptions.

^c^GINA class 1 is not applicable, since patients in class 1 only use SABA. ICS adherence is not applicable to those patients.

## Discussion

This study showed that both patients with an ICS adherence level of 50% or less and patients with an adherence level of over 90% were issued more SABA prescriptions. The number of SABA prescriptions was strongly associated with the occurrence of asthma exacerbations, whilst ICS adherence did not show a clear association. Patients with a higher number of SABA prescriptions in 2016 were more likely to have experienced one or more exacerbations in that year. Increased SABA use and an ICS adherence level of 50% or less were also strongly associated with self-reported uncontrolled asthma.

The SABA use of patients in our study appears to be similar to other European countries. A recent study investigating SABA overuse (at least three prescriptions per year) in the UK, Germany, Italy, Spain, and Sweden found percentages ranging from 9% in Italy up to 38% in the UK^25^. In our study, about 14% of patients received three or more SABA prescriptions.

In line with our study, an association between SABA use and exacerbations has been found in several studies^26,27^. More conflicting evidence has been found about the association between ICS adherence and exacerbations. Several studies have shown that a higher ICS adherence decreased the risk of exacerbations (e.g. refs. ^9,28^). However, like in our study, this association was not found in other studies^13,29^. It has been shown that ICS adherence fluctuates with an increase right before and after an exacerbation^28^. With our operationalization of ICS adherence (medication availability computed over the whole year using GP prescription data), these fluctuations could not be distinguished in the data. Our finding that self-reported uncontrolled asthma was associated with increased SABA use and low ICS adherence was in line with previous studies^29,30^.

The associations found in our study indicate a complex relation between controller and reliever medication use and exacerbations. On the one hand, there are patients who exhibit low ICS adherence (50% or less), and use more SABA (and who—independent of the ICS adherence level—also are at higher risk of experiencing exacerbations). These patients might either over-rely on their SABA for quick relief or they might confuse their controller and reliever medication. A recent study revealed that patients perceive their SABA as a great support in treating asthma symptoms driven by its immediate relief of symptoms^31^, and as such might prefer their SABA over their controller medication. This is also linked to the episodic nature of asthma; patients do not feel the need for long-term treatment with controller medication if they do not experience an impact on their daily life^32^. In addition, patients often do not realize that the frequent use of SABA indicates poorer asthma outcomes^31^. The combination of low ICS adherence and high SABA use might also be explained by patients’ confusion about their inhalers. Previous studies indicated that patients do not always know or understand the difference between their reliever medication and their controller medication to manage their asthma^32^. They use SABA regularly instead of their ICS medication unintentionally. A recent review showed similar or even better asthma symptom control and lower exacerbation rate in patients who use budesonide/formoterol as a maintenance and reliever therapy, compared to patients who use ICS/LABA with as-needed SABA^33^, also when used as as-needed medication (without maintenance)^34^. This as-needed combination medication, recommended for the first treatment step in the Netherlands since 2020^23^, can especially be suitable for patients with lower ICS adherence levels (and using SABA) or those who have a limited understanding of their asthma.

On the other hand, there are patients who are highly adherent to their controller medication (ICS adherence above 90%), but still use SABA often, and are as such also at higher risk for exacerbations.

Several factors may account for this behavior, including inhaler technique, adequacy of treatment, and exposure to environmental triggers. A correct technique is crucial for the effectiveness of ICS medication, i.e., if patients do not use their inhalers correctly, the medication does not reach its target and cannot be optimally effective. Patients thus might seem highly adherent as they “use” their medication according to the prescription, but do not benefit from the medication and are thus at higher risk for increased SABA use and exacerbations. Previous studies have revealed that many patients make critical errors in inhaling their medication, which has been shown to be associated with poor asthma outcomes^35,36^. Another factor might be the adequacy of the treatment. Patients might be treated with an inadequate dose of controller medication to control their asthma symptoms and therefore might more often need SABA to alleviate symptoms, whilst being adherent to the controller medication.

Finally, independent of the level of ICS adherence, patients might also require SABA frequently to regain control after being exposed to environmental triggers. Dima et al.’s Asthma Care Model (2016) includes, besides regular and correct use of maintenance medication, three other types of behavior patients need to perform for managing their asthma. These are self-monitoring of symptoms, management of triggers, and management of severe exacerbations^37^. Thus there can be several factors contributing to patients using SABA very frequently which cannot be disentangled easily from our study. However, the recently published study by Quint et al. (2022) also confirmed across other European countries that increasing SABA exposure is associated with increasing risk of exacerbation, independent of maintenance therapy^38^.

A strength of our study is that it used “real-world data”, i.e., data from a large primary care database. An advantage of these data is that they reflect daily practice. Using this type of data resulted in a large cohort of patients with asthma. After data preparation steps, we were provided with robust and interpretable data. It was necessary to exclude patients for whom we could not calculate adherence to controller treatment (23%); however, this was rather due to missing or incomplete information in the GP information system than to patient-related factors. The data preparation steps appeared to have had little impact on the representativeness of our study sample. The distribution of sex^39^, and percentage of patients per treatment step^40^ resembled the Dutch asthma population. Patients aged 12–17 years were somewhat underrepresented in our sample, as the Dutch asthma population is evenly distributed amongst age groups^41^. The average ICS adherence of 62% in our study sample was similar to the adherence levels found over the years 2007–2013 in the Netherlands, though these were based on dispensing data (www.TherapietrouwMonitor.nl) and similar to adherence levels found in other countries (e.g. refs. ^7,8^). SABA use in Dutch patients resembled SABA use in patients from other European countries^25^.

A limitation of our study is that our study period was limited to one year. Only exacerbations occurring in 2016 were included, thus disregarding whether patients have a short or long history with either few or many prior exacerbations. It would be interesting to investigate longer periods of time to investigate the effect of prior events. However, earlier studies already showed an association between consecutive exacerbations since each exacerbation causes irreversible damage to the lungs^42^. Another limitation is that data on healthcare utilization, such as emergency department visits, hospital admissions, or unscheduled GP visits, which often follow a severe exacerbation were not available. Short OCS courses with ≥20 mg daily were used as a proxy for exacerbations. This approach might have underestimated the number of exacerbations (capturing only mild to moderate exacerbations, and misclassifying patients with severe exacerbations to the reference group) which might have biased our results toward the null. Another limitation is that patients starting with ICS treatment in 2016 were not excluded from our sample. Patients who initiated treatment late in 2016 might have been overrepresented in the lowest adherence category, although two prescriptions were a minimum. In addition, we might have excluded patients with milder asthma as a result of having minimally two R03A and/or R03B prescriptions in 2016. We did not have detailed data on which the asthma diagnosis was based, we used the ICPC-code R96 to select patients with asthma. However, GPs follow their professional guideline “Asthma” in diagnosing patients which indicates that medical history should be assessed and physical examination and spirometry should be conducted.

The 227 practices were not fully representative of Dutch primary care, although the practices were located in both urban and rural areas, spread throughout the Netherlands, and the age distribution of our patient population resembled that of the total Dutch population.

There might also have been some residual confounding, other factors that play a role in the associations between ICS adherence, SABA use, and asthma outcomes besides the patient and clinical characteristics that were controlled for. For example, outcomes could have been influenced by factors such as environmental triggers (allergies, air pollution), whether ICS was correctly inhaled (inhaler technique) or whether all issued SABA prescriptions were actually used. SABA use may also be overestimated when patients have multiple inhalers which they keep at different locations for their convenience.

There are many ways to calculate adherence from administrative databases, all with their own strengths and limitations^43^ and each providing different estimates of adherence^44^. For this study, a CMA was used to operationalize adherence, more specifically the CMA7. The CMA7 takes carry-over into account from before the observation window as well as within the observation window. Disregarding the carry-over would underestimate the adherence rate. This is a clear advantage of CMA7. However, since CMA7 provides information about medication availability, overuse cannot be identified. Furthermore, prescription patterns are an estimate for medication adherence but lack information about actual intake. To actually monitor medication intake behavior, other adherence measures are necessary, e.g., electronic monitoring^45^.

The relation between ICS adherence and the number of SABA prescriptions with the risk of exacerbations is not a simple linear relationship. For GPs it is important to recognize that according to the SABA use of their patients with asthma, different approaches to achieving optimal asthma control are needed. Our study revealed that SABA use was associated with higher odds of exacerbations and higher odds of having self-reported uncontrolled asthma. Although the odds increased with the number of SABA prescriptions, patients having one or two prescriptions already were more likely to experience exacerbations. Yet, a higher number of SABA prescriptions was also associated with being more adherent to ICS. The GINA guidelines for treatment are updated annually and since 2019 recommend low-dose ICS or as-needed low-dose ICS/formoterol as the first step in treatment instead of SABA monotherapy. Our study supports these changes.

As GPs can easily identify patients with (higher) SABA use, compared to determining adherence to ICS medication, this should provide them with a clear signal to start the conversation with these patients about their asthma self-management. Our findings indicate that for achieving optimal asthma control, it is important for GPs to discuss with patients their asthma medication use (both controller and reliever medication), to check whether their inhaler technique is correct, and to determine whether the prescribed treatment is still adequate. Moreover, GPs should not only support patients in their medication use, but also support them in identifying and avoiding environmental triggers that worsen their symptoms. These implications endorse the 2020 updated Dutch guideline for GPs for the treatment of asthma in adults^23^.

In conclusion, SABA use was strongly associated with exacerbations, whereas ICS adherence was not. SABA use and poor ICS adherence were associated with self-reported uncontrolled asthma. These findings indicate that SABA use is an easily identifiable and important signal for GPs to discuss asthma management with their patients. To achieve better asthma outcomes, limiting SABA use, improving ICS adherence, optimizing treatment, but also other self-management behaviors, such as identifying and avoiding triggers, need to be considered.

## Supplementary information

Supplementary material

REPORTING SUMMARY

## Data Availability

The dataset generated and analyzed during the current study and the codes are available from the corresponding author on reasonable request.
