# Supplementary material for: Understanding relationships between asthma medication use and outcomes in a SABINA primary care database study
Source: NPJ Prim Care Respir Med. 2022 Oct 21;32:43. doi: 10.1038/s41533-022-00310-x (PMC9587241; doi:10.1038/s41533-022-00310-x)
Supplement: Supplementary file 1 — Supplementary material [file 41533_2022_310_MOESM1_ESM.docx]

**Supplementary information**

*Supplementary table 1. List of comedication*

| **Medication** | **ATC-codes** |
| --- | --- |
| Antihistaminergic agents | R06 |
| Nasal decongestants | R01B |
| Systemic antibiotics | J01 |
| Systematic corticosteroids | H02 (excl. H02AB06 & H02AB07) |
| Beta blockers | C07 |
| Cardiac drugs | C01 |
| Antidiabetic drugs | A10 |
| NSAIDs | M01A |
| Paracetamol | N02BE01 |
| GERD drugs | A03B |
| Tricyclic antidepressants | N06AA |
| Other antidepressants | N06A (other than N06AA) |

*Supplementary table 2. List of comorbidities*

| **No** | **Chronic disease** | **ICPC-1^#^ codes** |
| --- | --- | --- |
| 1 | Hypertension | K86, K87 |
| 2 | Diabetes Mellitus | T90 |
| 3 | Osteoarthritis | L89-L91 |
| 4 | Coronary artery disease | K74-K76 |
| 5 | Cancer | A79, B72-B74, D74-D77, L71, N74, R84, R85, S77, T71, U75‑U77, W72, X75-X77, Y77, Y78 |
| 6 | Chronic back or neck disorder | L83, L84, L86 |
| 7 | COPD* | R91, R95 |
| 8 | Visual disorder | F83, F84, F92-F94 |
| 9 | Cardiac dysrhythmia | K78-K80 |
| 10 | Depression (and psychosis) | P73, P76 |
| 11 | Heart failure | K77 |
| 12 | Asthma* | R96 |
| 13 | Hearing disorder | H84-H86 |
| 14 | Osteoporosis | L95 |
| 15 | Stroke | K90 |
| 16 | Rheumatoid arthritis | L88 |
| 17 | Dementia (incl. Alzheimer’s disease) | P70 |
| 18 | Anxiety disorder | P74 |
| 19 | Migraine | N89 |
| 20 | Heart valve disorder | K83 |
| 21 | Neuraesthenia/surmenage/burn-out | P78 |
| 22 | Chronic alcohol abuse | P15 |
| 23 | Parkinson’s disease | N87 |
| 24 | Epilepsy | N88 |
| 25 | Personality disorder | P80 |
| 26 | Schizophrenia | P72 |
| 27 | Intellectual disability | P85 |
| 28 | Congenital cardiovascular anomaly | K73 |
| 29 | HIV/AIDS | B90 |

** Excluded from analyses*

*Supplementary table 3. Description of how medication regimes in this study were labelled to the GINA classification of 2016*

| Step 1 | Step 2 | Stap 3 | Stap 4 | Stap 5 |
| --- | --- | --- | --- | --- |
| **Only SABA/SAMA** ‘as needed’  - salbutamol  (R03AC02)  - terbutaline  (R03AC03)  - ipratropium (R03BB01) | **Low dose ICS**  - beclometasone (R03BA01): 100-200 mcg  - budesonide (R03BA02): 200-400 mcg  - fluticasone furoate (R03BA09): 100 mcg  - fluticasone proprionate (R03BA05): 100-250 mcg  - ciclesonide (R03BA08): 80-160 mcg | **Low dose ICS/LABA**  - beclometasone/formoterol (R03AK08): 100-200 mcg  - budesonide/formoterol (R03AK07): 200-400 mcg  - budesonide/salmeterol (R03AK12): 200-400 mcg  - fluticasone/salmeterol (R03AK06): 100-250 mcg  - fluticasone/formoterol (R03AK11): 100-250 mcg  LABA with low dose ICS:  - salmeterol (R03AC12)  - formoterol (R03AC13) | **Medium/high dose ICS/LABA**  - beclometasone/ formoterol (R03AK08): >200 mcg  - budesonide/formoterol (R03AK07): >400 mcg  - budesonide/salmeterol (R03AK12): >400 mcg  - fluticasone/salmeterol (R03AK06): >250 mcg  - fluticasone/formoterol (R03AK11): >250 mcg  LABA with medium/high dose ICS (see step 3):  - salmeterol (R03AC12)  - formoterol (R03AC13) | **Add tiotropium mist inhaler** (R03BB04 product number 84808 or 128201) |
|  | **LTRA**  Only LTRA, without ICS  - montelukast (R03DC03) | **Low dose ICS/LABA + LTRA** | **Medium/high dose ICS/LABA + tiotropium** | **Add anti-IgE**  - omalizumab (R03DX05) |
|  | **Low dose theophylline^1^**  Only theophylline, without ICS  - theophylline (R03DA04) | **Low dose ICS/LABA + theophylline** | **Medium/high dose ICS + LTRA** | **Add anti-IL5**  - mepolizumab (R03DX09) |
|  |  | **Low dose ICS + LTRA** | **Medium/high dose ICS + theophylline** | **Add low dose OCS**:  <20mg prednisolone (H02AB06) or prednisone  (H02AB07) |
|  |  | **Low dose ICS + theophylline** |  |  |
|  |  | **Medium/high dose ICS**  - beclometasone (R03BA01):  >200 mcg  - budesonide (R03BA02):  >400 mcg  - fluticasone furoate (R03BA09): 200 mcg  - fluticasone proprionate (R03BA05): >250 mcg  - ciclesonide (R03BA08): >160 mcg |  |  |

*Supplementary table 4. Characteristics of the study population (N=13,756) compared to those for whom no ICS adherence rate could be calculated (N=5,873)*

|  | **Patients for whom ICS adherence was calculated**  **(N=13,756)** | **Patients for whom no ICS adherence could be calculated (N=5,873)** |
| --- | --- | --- |
|  | **n (%)** | **n (%)** |
| **Sex^*^** |  |  |
| Male | 5,531 (40.2) | 2,264 (38.6) |
| Female | 8,225 (59.8) | 3,609 (61.5) |
| **Age^***^(years)** |  |  |
| 12-17 | 981 (7.1) | 331 (5.6) |
| 18-39 | 2,919 (21.2) | 1,217 (20.7) |
| 40-54 | 3,800 (27.6) | 1,637 (27.9) |
| 55-64 | 2,692 (19.6) | 1,115 (19.0) |
| 65+ | 3,364 (24.5) | 1,573 (26.8) |
| **Comedication^*^** |  |  |
| 0 | 1,618 (11.8) | 629 (10.7) |
| 1 | 2,768 (20.1) | 1,116 (19.0) |
| 2 | 2,978 (21.7) | 1,291 (22.0) |
| >2 | 6,392 (46.5) | 2,837 (48.3) |
| **Comorbidity** |  |  |
| 0 | 4,945 (36.0) | 2,054 (35.0) |
| 1 | 3,146 (22.9) | 1,381 (23.5) |
| 2 | 2,158 (15.7) | 942 (16.0) |
| >2 | 3,507 (25.5) | 1,496 (25.5) |
| **GINA class^***^** | **(n=13,694)** | **(n=5,035)** |
| 2^#^ | 1,743 (12.7) | 817 (16.2) |
| 3 | 4,886 (35.7) | 1,957 (38.9) |
| 4 | 6,672 (48.7) | 2,100 (41.7) |
| 5 | 393 (2.9) | 161 (3.2) |
| **ICS adherence** |  |  |
| ≤50% | 5,488 (39.9) | N/A |
| 51-60% | 1,084 (7.9) | N/A |
| 61-70% | 1,025 (7.5) | N/A |
| 71-80% | 990 (7.2) | N/A |
| 81-90% | 783 (5.7) | N/A |
| 91-100% | 4,386 (31.9) | N/A |
| **SABA prescriptions^**^** |  |  |
| 0 | 5,916 (43.0) | 2,773 (47.2) |
| 1-2 | 5,957 (43.4) | 2,257 (38.4) |
| 3-6 | 1,600 (11.6) | 718 (12.2) |
| 7-12 | 233 (1.7) | 104 (1.8) |
| ≥13 | 50 (0.4) | 21 (0.4) |
| **Exacerbations** |  |  |
| 0 | 11,947 (86.9) | 5,123 (87.2) |
| 1 | 1,387 (10.1) | 574 (9.8) |
| 2 | 318 (2.3) | 140 (2.4) |
| 3 | 67 (0.5) | 23 (0.4) |
| ≥4 | 37 (0.3) | 13 (0.2) |

*^#^ GINA class 1 is not applicable, since patients in class 1 only use SABA and no ICS*

** p<.05; ** p<0.01; *** p<.001*

*Supplementary table 5. Characteristics of the study sample for whom an ACQ-5 score was available compared to those for whom no ACQ-5 score was available*

|  | **Patients for whom an**  **ACQ-5 score was available**  **(N=2,183)** | **Patients for whom**  **no ACQ-5 score was available**  **(N=11,573)** |
| --- | --- | --- |
|  | **n (%)** | **n (%)** |
| **Sex** |  |  |
| Male | 849 (38.9) | 4,682 (40.5) |
| Female | 1,334 (61.1) | 6,891 (59.5) |
| **Age^***^ (years)** |  |  |
| 12-17 | 119 (5.5) | 862 (7.5) |
| 18-39 | 449 (20.6) | 2,470 (21.3) |
| 40-54 | 562 (25.7) | 3,238 (28.0) |
| 55-64 | 448 (20.5) | 2,244 (19.4) |
| 65+ | 605 (27.7) | 2,759 (23.8) |
| **Comedication** |  |  |
| 0 | 239 (11.0) | 1,379 (11.9) |
| 1 | 415 (19.0) | 2,353 (20.3) |
| 2 | 500 (22.9) | 2,478 (21.4) |
| >2 | 1,029 (47.1) | 5,363 (46.3) |
| **Comorbidity** |  |  |
| 0 | 737 (33.8) | 4,208 (36.4) |
| 1 | 517 (23.7) | 2,629 (22.7) |
| 2 | 370 (17.0) | 1,788 (15.5) |
| >2 | 559 (25.6) | 2,948 (25.5) |
| **GINA class^***^** | **(n=2,174)** | **(n=11,520)** |
| 2^#^ | 249 (11.5) | 1,494 (13.0) |
| 3 | 830 (38.2) | 4,056 (35.2) |
| 4 | 1,056 (48.6) | 5,616 (48.8) |
| 5 | 39 (1.8) | 354 (3.1) |
| **ICS adherence^***^** |  |  |
| ≤50% | 616 (28.2) | 4,872 (42.1) |
| 51-60% | 187 (8.6) | 897 (7.8) |
| 61-70% | 155 (7.1) | 870 (7.5) |
| 71-80% | 142 (6.5) | 848 (7.3) |
| 81-90% | 125 (5.7) | 658 (5.7) |
| 91-100% | 958 (43.9) | 3,438 (29.6) |
| **SABA prescriptions** |  |  |
| 0 | 985 (45.1) | 4,931 (42.6) |
| 1-2 | 934 (42.8) | 5,023 (43.4) |
| 3-6 | 226 (10.4) | 1,374 (11.9) |
| 7-12 | 31 (1.4) | 202 (1.8) |
| ≥13 | 7 (0.3) | 43 (0.4) |
| **Exacerbations** |  |  |
| 0 | 1,917 (87.8) | 10,030 (86.7) |
| 1 | 215 (9.9) | 1,172 (10.1) |
| 2 | 42 (1.9) | 276 (2.4) |
| 3 | 6 (0.3) | 61 (0.5) |
| ≥4 | 3 (0.1) | 34 (0.3) |

*^#^ GINA class 1 is not applicable, since patients in class 1 only use SABA and no ICS*

** p<.05; ** p<0.01; *** p<.001*

**TEOS framework for operationalizing adherence concepts**

Supplementary table 6: TEOS operationalization for ICS adherence in this study

| Timelines: | **Prescription events:** in Dutch primary care, ICS prescriptions are issued usually for three months (D_1_) and renewed during follow-up consultations (D_2…_); prescription duration may vary. Treatment is prescribed long-term without interruptions; it may be stepped up or stepped down or (rarely) discontinued by the prescriber.  **Recommended dosing events:** treatment starts immediately after the first ICS prescription; dosing is daily; recommendations may vary within and between patients, and between prescribers. Dosing adjustment may sometimes be recommended to patients.  **Dispensing events:** dispensing events start within 30 days of/immediately after the first prescription date and cover between 30 and 90 days (D_3,_ D_4…_).  **Actual dosing events:** ICS are administered by inhalation, effectiveness depends on inhaler technique; variations in adherence may consist of taking less or more inhalations, skip or change an administration time, or administer incorrectly. |
| --- | --- |
| Events: | The study selects patients with at least two years of data in the primary care database (one baseline year and one follow-up year), and at least two prescriptions of inhalation medication in the ATC groups R03A and R03B during the follow-up year.  **First recommended dosing event:** date of first prescription (T_0_) occurs before or during the follow-up year.  **Last recommended dosing event:** It is assumed that for the sample selected based on two or more prescriptions there was no gap of more than six months during the follow-up year to indicate end of prescription.  **First actual dosing event:** occurs after first dispensation (T_1_)  **Last actual dosing event:** assumed based on estimated end of prescribed supply given dosing recommendations, likely to occur after the end of the follow-up year as only patients having at least two prescriptions during 2016 were selected (T_2_) |
| Objectives: | We are interested in between person variation in ICS adherence and its effects on **asthma exacerbations** and **self-reported** **asthma control**, also in relation to SABA use and other socio-demographic and clinical covariates. Asthma control varies in time under the influence of multiple factors. There are likely bidirectional relationships between asthma control and asthma exacerbations and ICS adherence and SABA use. Asthma exacerbations and asthma control may vary **between patients**, **healthcare** **professionals**, and **organisations**. This variation may be partly due to variations in usual care at **General Practioner** and **primary care practice** levels. |
| Sources: | **Prescription** data (dates, dosing recommendations, prescribed amount) are manually entered by the general practitioners in the practices participating in the Nivel Primary Care Database. **Dispensing** data are not available. **Electronic monitoring** is not feasible at this scale and rarely used in routine practice long-term, as is self-report of adherence. |

*Supplementary box 1: Operational definitions for measuring adherence*

**Initiation**: Assumed 1st prescription was before Jan 2016

**Persistence**: Not studied

**Implementation**: Quality of implementation calculated from prescription data.

Implementation was operationalized with a Continuous multiple-interval measure of Medication Availability (CMA7). CMA7 is calculated by dividing the number of days of theoretical use by the number of days between start to end of the observation window (OW) (366 days in 2016). Days of theoretical use are calculated by extracting the total number of gap days (days for which no medication is available) from the total number of days of the OW, accounting for carry-over for all prescriptions within and before the OW.

Quantification: Continuous variable (percentage, range 0 – 100), categorical variable with six categories : ≤50%, 51-60%, 61-70%, 71-80%, 81-90%, 91-100%.

Summary measure: mean implementation score (SD); frequencies of categories.

Supplementary
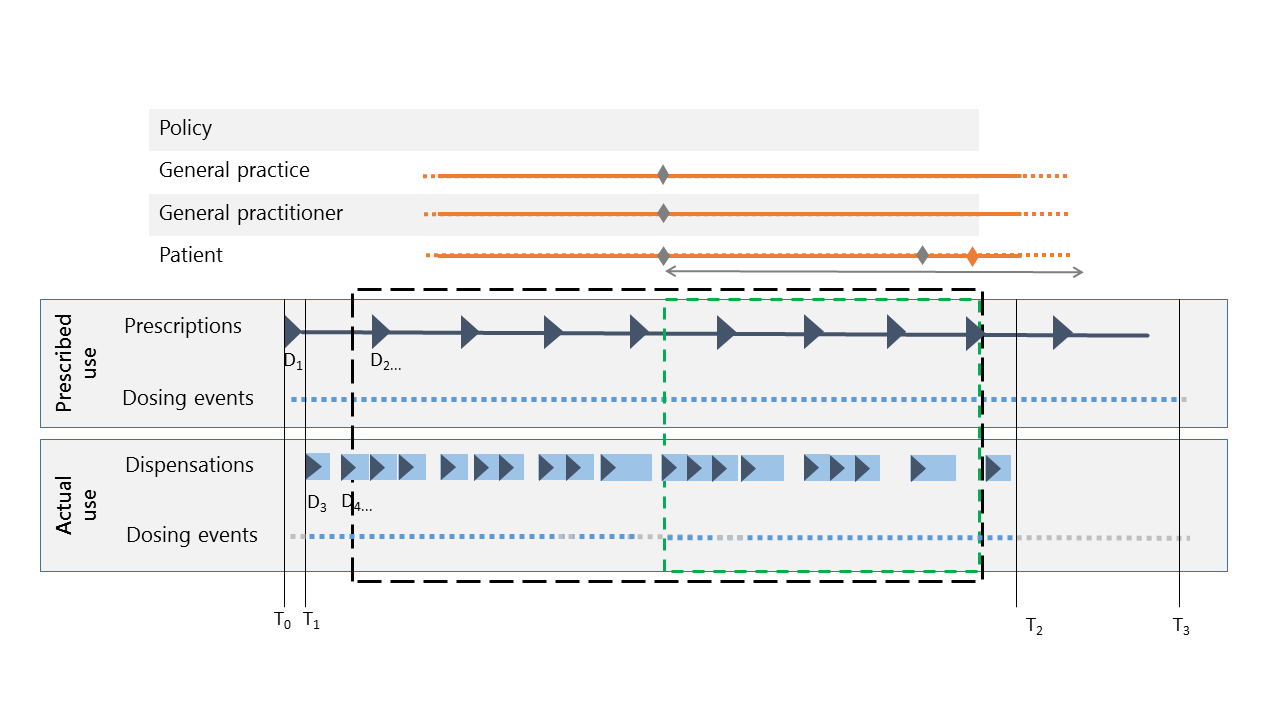
figure 1: Graphical representation of the TEOS operationalization of ICS adherence.

D_1_-D_4_ and T_0_-T_3_ refer to the above-mentioned timelines and events, respectively. Dashed rectangles: adherence calculation follow-up window (black) and observation window (green). Usual care (orange line) is provided by general practitioners to patients within different practices. Adherence is measured at patient level (orange lozenges) once. Additional variables are measured at inclusion at patient level; self-reported asthma control may be recorded at different times during the observation window or during the 92 days after (grey lozenges).
