# Supplementary material for: Understanding relationships between asthma medication use and outcomes in a SABINA primary care database study
Source: NPJ Prim Care Respir Med. 2022 Oct 21;32:43. doi: 10.1038/s41533-022-00310-x (PMC9587241; doi:10.1038/s41533-022-00310-x)
Supplement: Supplementary file 2 — REPORTING SUMMARY [file 41533_2022_310_MOESM2_ESM.pdf]

nature portfolio

Corresponding author(s): Marcia Vervloet

Last updated by author(s): Jun 3, 2022

Reporting Summary

Nature Portfolio wishes to improve the reproducibility of the work that we publish. This form provides structure for consistency and transparency in reporting. For further information on Nature Portfolio policies, see our Editorial Policies and the Editorial Policy Checklist.

Statistics

For all statistical analyses, confirm that the following items are present in the figure legend, table legend, main text, or Methods section.

n/a	Confirmed	
☐	☒	The exact sample size (*n*) for each experimental group/condition, given as a discrete number and unit of measurement
☐	☒	A statement on whether measurements were taken from distinct samples or whether the same sample was measured repeatedly
☐	☒	The statistical test(s) used AND whether they are one- or two-sided<br>*Only common tests should be described solely by name; describe more complex techniques in the Methods section.*
☐	☒	A description of all covariates tested
☐	☒	A description of any assumptions or corrections, such as tests of normality and adjustment for multiple comparisons
☐	☒	A full description of the statistical parameters including central tendency (e.g. means) or other basic estimates (e.g. regression coefficient) AND variation (e.g. standard deviation) or associated estimates of uncertainty (e.g. confidence intervals)
☐	☒	For null hypothesis testing, the test statistic (e.g. *F*, *t*, *r*) with confidence intervals, effect sizes, degrees of freedom and *P* value noted<br>*Give P values as exact values whenever suitable.*
☒	☐	For Bayesian analysis, information on the choice of priors and Markov chain Monte Carlo settings
☐	☒	For hierarchical and complex designs, identification of the appropriate level for tests and full reporting of outcomes
☒	☐	Estimates of effect sizes (e.g. Cohen's *d*, Pearson's *r*), indicating how they were calculated

Our web collection on statistics for biologists contains articles on many of the points above.

Software and code

Policy information about availability of computer code

Data collection	We used STATA SE16 for data collection
Data analysis	We used STATA SE16 for data analysis

For manuscripts utilizing custom algorithms or software that are central to the research but not yet described in published literature, software must be made available to editors and reviewers. We strongly encourage code deposition in a community repository (e.g. GitHub). See the Nature Portfolio guidelines for submitting code & software for further information.

Data

Policy information about availability of data

All manuscripts must include a data availability statement. This statement should provide the following information, where applicable:

- Accession codes, unique identifiers, or web links for publicly available datasets
- A description of any restrictions on data availability
- For clinical datasets or third party data, please ensure that the statement adheres to our policy

The dataset generated and analysed during the current study and the codes are available from the corresponding author on reasonable request.

Human research participants

Policy information about studies involving human research participants and Sex and Gender in Research.

Reporting on sex and gender	Sex is recorded in the primary care database. In all analyses, sex is included as a variable. we found that being female significantly increased the odds of having one or more exacerbations. Women were also less likely to report controlled asthma.
Population characteristics	Patients aged 12 years and older, diagnosed with asthma (ICPC-code R96), who received at least two prescriptions of inhalation medication in ATC groups R03A (adrenergics) and/or R03B (glucocorticoids and anticholinergics) in 2016 were selected. Patients who were also diagnosed with chronic obstructive pulmonary disease (COPD, ICPC-code R95) in 2016 were excluded.
Recruitment	We used routinely collected data for primary care practices. Data from practices and patients who fulfilled our inclusion criteria were extracted. The following criteria per practice were met: a minimum of 500 patients (the average number of patients of 1 fte GP in the Netherlands is 2095), a minimum of 46 weeks of registration per year, complete data for the period 2015-2017 (necessary for adherence calculations), and illness episodes could be constructed (necessary for determining morbidities)18. As a result, a total of 227 general practices were included in this study. Then, patients aged 12 years and older, diagnosed with asthma (ICPC-code R96), who received at least two prescriptions of inhalation medication in ATC groups R03A (adrenergics) and/or R03B (glucocorticoids and anticholinergics) in 2016 were selected. Patients who were also diagnosed with chronic obstructive pulmonary disease (COPD, ICPC-code R95) in 2016 were excluded.
Ethics oversight	This study has been approved according to the governance code of Nivel Primary Care Database, under number NZR-00318.050

Note that full information on the approval of the study protocol must also be provided in the manuscript.

Field-specific reporting

Please select the one below that is the best fit for your research. If you are not sure, read the appropriate sections before making your selection.

☐ Life sciences ☒ Behavioural & social sciences ☐ Ecological, evolutionary & environmental sciences

For a reference copy of the document with all sections, see nature.com/documents/nr-reporting-summary-flat.pdf

Behavioural & social sciences study design

All studies must disclose on these points even when the disclosure is negative.

Study description	Database study
Research sample	Our sample consisted of 13,756 patients with asthma. Six out of ten patients were female. About 7% of the patients were adolescents, whereas the largest age-category was 40-54 years (28%), and nearly a quarter of the patients were 65 years or older. Most patients (88%) used other chronic medication besides their asthma medication and almost two-third of patients suffered from other chronic illnesses besides asthma. More than four out of ten patients were treated according to GINA step 4. ICS adherence averaged 62% (SD: 32.7). About 40% of patients had an ICS adherence level of 50% or lower, while almost a third had an adherence level of more than 90%. The majority of patients (86%) were issued two or fewer SABA prescriptions in 2016, 14% had three or more prescriptions. The average number of SABA prescriptions was 1.2 (SD: 1.8). The majority of patients (87%) experienced no exacerbations.
Sampling strategy	This is not applicable for our database study
Data collection	Data were extracted from the Nivel Primary Care Database, which includes routine care data originating from electronic medical records from about 500 general practices (10% of all practices) across the Netherlands. These practices constitute a representative sample of the total population of Dutch general practices. Within the Dutch health care system all residents are mandatorily registered with one general practitioner (GP), who coordinates their care. The database includes information on patient characteristics (age, sex), time-stamped GP consultations, diagnoses and medication prescriptions. Diagnoses are recorded by GPs using the International Classification of Primary Care version 1 (ICPC-1). Prescriptions are coded using the Anatomical Therapeutic Chemical Classification system (ATC). Only coded data were extracted, not free text.
Timing	Data were extracted for 2015-2017, with the year of interest being 2016
Data exclusions	This is not applicable for our database study
Non-participation	This is not applicable for our database study

Randomization	This is not applicable for our database study

Reporting for specific materials, systems and methods

We require information from authors about some types of materials, experimental systems and methods used in many studies. Here, indicate whether each material, system or method listed is relevant to your study. If you are not sure if a list item applies to your research, read the appropriate section before selecting a response.

Materials & experimental systems

n/a	Involved in the study
☒	☐ Antibodies
☒	☐ Eukaryotic cell lines
☒	☐ Palaeontology and archaeology
☒	☐ Animals and other organisms
☒	☐ Clinical data
☒	☐ Dual use research of concern

Methods

n/a	Involved in the study
☒	☐ ChIP-seq
☒	☐ Flow cytometry
☒	☐ MRI-based neuroimaging
